# Data-adaptive image-denoising for detecting and quantifying nanoparticle entry in mucosal tissues through intravital 2-photon microscopy

**DOI:** 10.3762/bjnano.5.210

**Published:** 2014-11-06

**Authors:** Torsten Bölke, Lisa Krapf, Regina Orzekowsky-Schroeder, Tobias Vossmeyer, Jelena Dimitrijevic, Horst Weller, Anna Schüth, Antje Klinger, Gereon Hüttmann, Andreas Gebert

**Affiliations:** 1 University Hospital Jena, Friedrich Schiller University Jena, Institute of Anatomy II, Teichgraben 7, 07740 Jena, Germany; 2 University of Lübeck, Institute of Biomedical Optics, Peter-Monnik-Weg 4, 23562 Lübeck, Germany; 3 Olympus Winter & Ibe GmbH, R&D Optical Design, Kuehnstrasse 61, 22045 Hamburg, Germany; 4 University of Hamburg, Institute of Physical Chemistry, Grindelallee 117, 20146 Hamburg, Germany; 5 University of Lübeck, Institute of Anatomy, Ratzeburger Allee 160, 23538 Lübeck, Germany

**Keywords:** 2-photon microscopy (2PM), denoising, in vivo imaging, nanoparticles, signal to noise ratio (SNR), quantum dots

## Abstract

Intravital 2-photon microscopy of mucosal membranes across which nanoparticles enter the organism typically generates noisy images. Because the noise results from the random statistics of only very few photons detected per pixel, it cannot be avoided by technical means. Fluorescent nanoparticles contained in the tissue may be represented by a few bright pixels which closely resemble the noise structure. We here present a data-adaptive method for digital denoising of datasets obtained by 2-photon microscopy. The algorithm exploits both local and non-local redundancy of the underlying ground-truth signal to reduce noise. Our approach automatically adapts the strength of noise suppression in a data-adaptive way by using a Bayesian network. The results show that the specific adaption to both signal and noise characteristics improves the preservation of fine structures such as nanoparticles while less artefacts were produced as compared to reference algorithms. Our method is applicable to other imaging modalities as well, provided the specific noise characteristics are known and taken into account.

## Introduction

Imaging methods applied to detect fluorescent nanoparticles in mucosal tissues should provide high optical resolution and allow large volumes to be scanned. An important and versatile tool for this purpose is intravital 2-photon microscopy (2PM) based on tissue autofluorescence [1]. Volumes of up to 600 × 600 × 150 µm^3^ can be scanned within less than 30 seconds, and repeated scans allow for individual particles to be tracked over minutes or even hours. However, digital images recorded under such conditions typically contain large amounts of noise, i.e., statistical variations of the pixel intensities that do not correspond to tissue structures.

The signal to noise ratio (SNR) cannot readily be increased by slower scanning or binning, because this would critically affect the temporal or spatial resolution required for particle tracking. Increased intensities of the excitation light might also improve the SNR, but phototoxic damage closely limits the amount of light that can be applied to living cells. Intravital 2PM at fast frame rates is thus a low light method in which, at least in dark image areas, only very few photons are collected per pixel. This unavoidably leads to a low SNR, which not only affects further data interpretation by human observers, but also deteriorates the efficiency of automated processing, segmentation and image analysis [2–4].

In our approach, raw image data are digitally processed to reconstruct an estimation of the underlying ground-truth signal by suppressing the noise. This so-called denoising is a typical task in signal processing, for which, in the last decades, numerous methods were developed [5–10]. A few modern denoising methods have already been adapted to biomedical data sets recorded at low photon counts [11–13]. However, typical limitations and drawbacks of denoising methods comprise flattening of edges, the production of artefacts, and the removal of significant image details [14]. To overcome especially the latter remains a challenging task in quantitative nanoparticle studies. We first evaluated the suitability of established denoising methods and then improved the best suited method by adaptation to both signal and noise characteristics of our specific application.

### Formal description of the imaging process

In the following, we will refer to the underlying ground-truth image as 
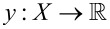
. Thereby the image domain is referred to as 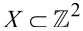, and 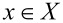 is a spatial coordinate that belongs to the image. Because of the unavoidable noise, we cannot measure *y* itself but the noisy image *z.* Considering the noise as η, the imaging process is described by:

[1]
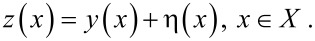


### Existing methods tested for denoising

**Figure 1:**
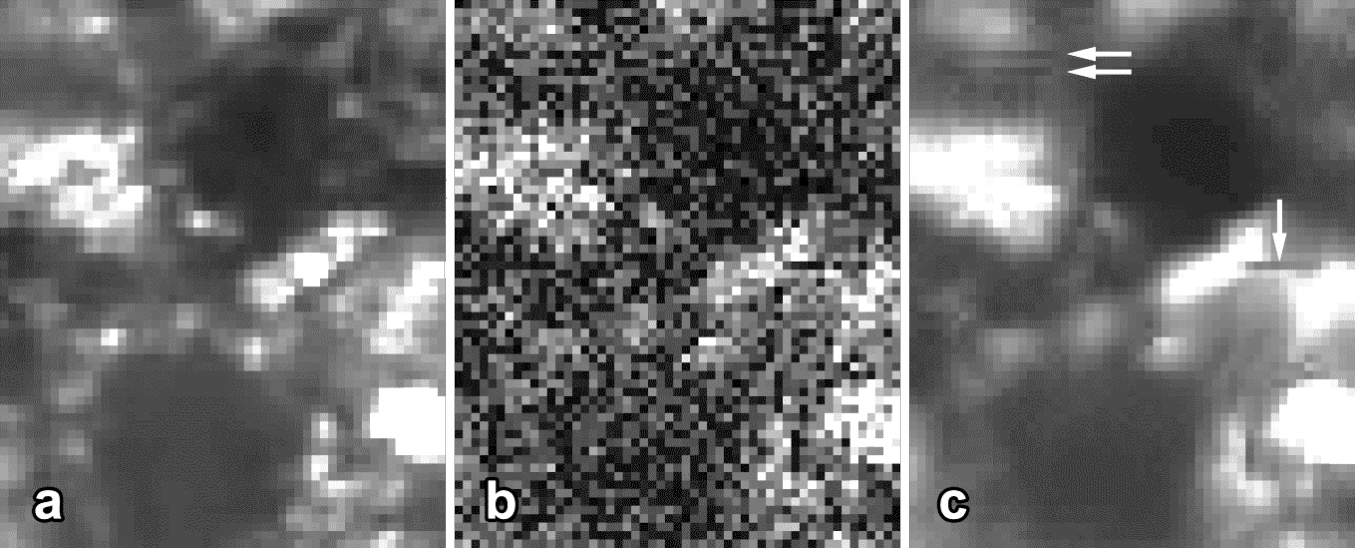
Enlarged optical section of gut tissue in intravital 2-photon microscopy: a) ground-truth signal, b) ground truth with digitally added noise, c) image reconstructed by the original BM3D algorithm published by Dabov et al. [15]. Note that the reconstructed image contains a number of structures which are not seen in the original image and thus represent artefacts (arrows).

We investigated the usability of three advanced denoising methods in case of low-photon-counts nanoparticle imaging. These procedures, named BM3D [15], SAFIR-nD [11], and PURE-LET [12], have previously been compared to other modern denoising methods [10,16–18] and therefore can be considered state of the art in denoising. Preliminary tests (see Supporting Information File 1, Figure S1) revealed that the BM3D algorithm [15] applied to 2PM images generated the best results regarding general image definition, but still produced some artefacts, flattened edges, and removal of some fine image details (Figure 1). As the general strategy of the BM3D algorithm was used as a starting point for our work it will be described briefly in the following.

### Principles of the BM3D denoising method

In general, the strategy of the BM3D algorithm exploits the fact that many images, including microscopic views of biomedical structures, typically contain redundant information. Cells or organelles of the same type possess similar shapes, outlines, curvatures and grayscale distributions, and thus share common morphological features. The algorithm exploits both local and non-local redundancy of *y*(*x*) by grouping similar image blocks to three-dimensional arrays and jointly filtering of these stacks. The grouping is made by block-matching while the filtering and suppression of noise is accomplished by enforcing sparsity of the coefficients in the 3D-transform domain.

**Figure 2:**
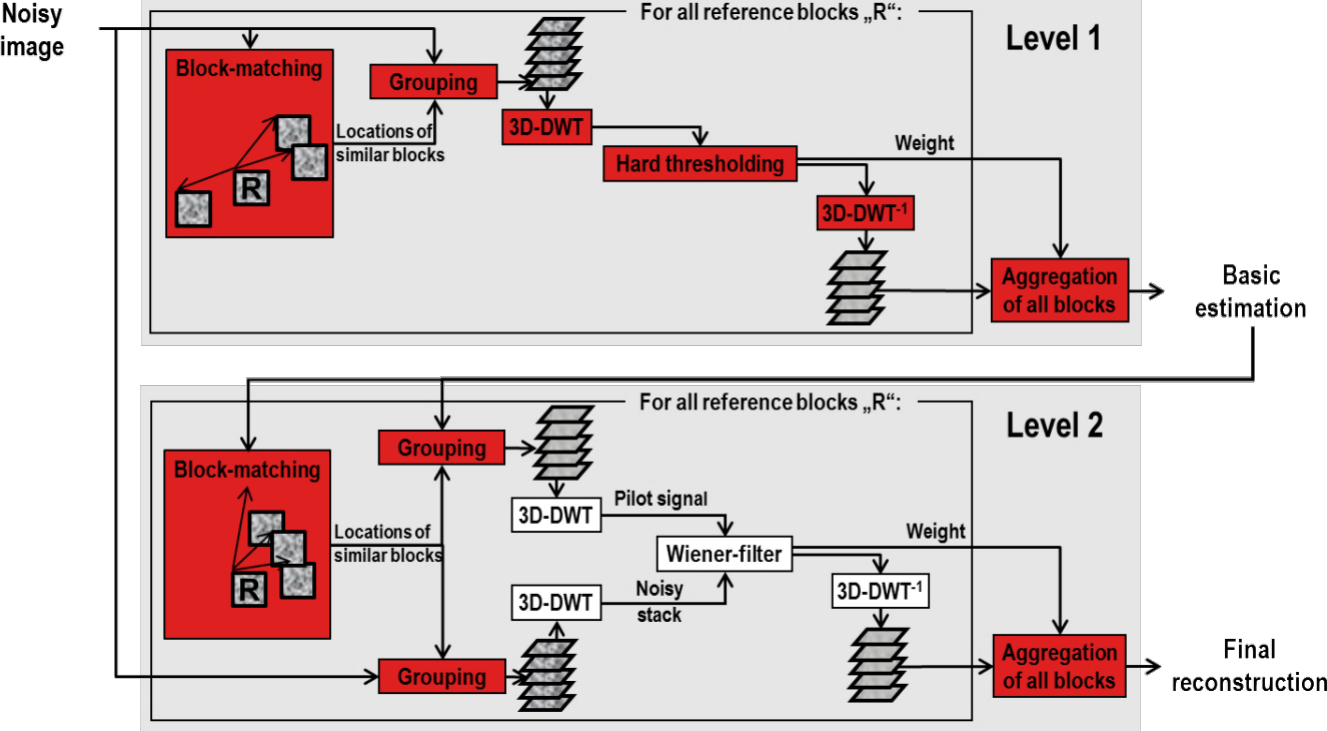
Flowchart of the BM3D algorithm. Red-colored steps were modified by us in order to adapt the algorithm described by Dabov et al. [15] to nanoparticle detection in mucosal tissues. DTW, discrete wavelet transform.

The basic algorithm is illustrated in Figure 2 and consists of two iterative levels (see [15] for details):

**Level 1: Basic estimation** 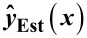 **of the ground-truth signal**

1. For each reference block (e.g., 8 × 8 pixels sliding over the whole image in steps of 3 pixels) in *z*(*x*):
  *Grouping:* Find blocks that are similar to the currently processed reference block and group them to a stack.
  *Collaborative hard-thresholding:* 1. Transform the stack to a sparse representation domain (e.g., by using a discrete wavelet transform (DWT)); 2. Suppress the noise by hard-thresholding, i.e., set all coefficients below the threshold to zero; 3. Invert the transform.
  Return all stacked blocks to their original positions.
2. Aggregation: Compute 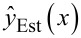 by weighted averaging of all block-wise estimates that are overlapping.

**Level 2: Final reconstruction by using** 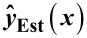 **for improved block-matching and as pilot signal for collaborative Wiener-filtering**

1. For each reference block in the measured signal *z*(*x*)*:*
  *Grouping:* Perform the block-matching within 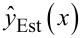 to find locations of blocks that are similar to the currently processed reference block. By using these locations, group the blocks to two stacks, one from *z*(*x*) and one from 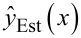.
  *Collaborative Wiener-filtering:* 1. Apply a transform to a sparse representation domain on both groups; 2. Perform Wiener-filtering on the stack from *z*(*x*) by using the stack from 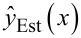 as the pilot signal; 3. Invert the transform.
  Return all filtered blocks to their original positions.
2. Aggregation: Compute the final reconstruction 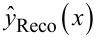 by weighted averaging of all filtered blocks that are overlapping.

### Adaptation of the BM3D algorithm to low-photon-counts imaging

Due to the particle nature of light, the number of detected photons has a Poisson distribution that depends on *y*(*x*). For low photon counts such fluctuations, also called shot noise, cannot be described properly as additive white Gaussian noise (AWGN), which is independent of the signal. A more generic noise model of the form:

[2]
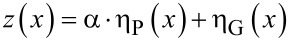


has to be used, in which η_p_(*x*) ~ *P*(*y*(*x*)) is a Poisson-distributed signal-dependent component, scaled by a constant α > 0 and η_G_(*x*) a Gaussian-distributed signal-independent component with mean μ_G_ and variance σ_G_^2^.

BM3D had originally been designed for denoising images affected by AWGN. Therefore, the noise variance has to be stabilized by the generalized Anscombe root transformation [19] applied to *z*(*x*). In the resulting signal, the noise can be regarded as AWGN, and efficiently suppressed by applying BM3D. Finally, the inverse transform has to be applied to obtain the reconstructed image. This was done by the exact inverse transform, because it produces less reconstruction errors as compared to the algebraic and the asymptotically unbiased inverse transform [19].

## Results and Discussion

### Validation of the noise model

Noise measurements of homogeneous fluorescent plastic slices in 2PM were performed to verify the noise model (see Experimental). While the ground-truth signal of these samples is constant, the resulting image intensities are not because of the noise. For this reason we can estimate the noise intensities by subtracting the mean image intensity from the measured intensity values. Considering the cardinality of *X* as |*X*|, the noise is described by:

[3]
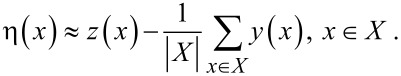


**Figure 3:**
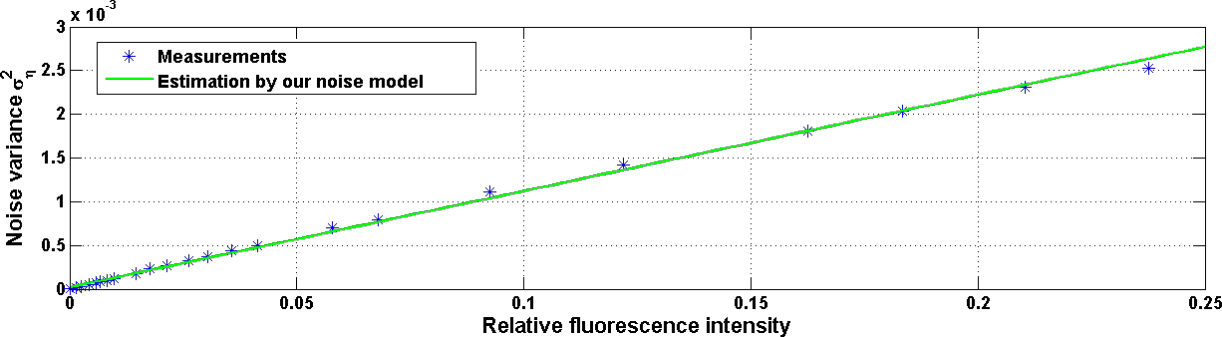
Verification of the noise model: comparison of noise variance measurements (σ_η_^2^) with estimations proposed by the noise model. The data show that the noise model closely fits the 2PM measurements.

By using this approximation of η(*x*) we estimated the variance of the noise, σ_η_^2^, for the whole series of noise measurements and thus for different values of excitation laser power. Plotting σ_η_^2^ against the fluorescence intensity reveals a linear dependency between the two quantities and clearly shows the heteroscedasticity of the noise (see below in Figure 3).

It follows from the foregoing that, under typical conditions of intravital 2PM, the noise variance cannot properly be described by a signal-independent model. Instead a signal-dependent model as the proposed Poisson–Gaussian mixture model has to be used to correctly describe the heteroscedastic noise. Therefore, as described in the foregoing section, the generalized Anscombe root transformation [19] was applied to *z*(*x*) in order to stabilize σ_η_^2^.

For further verification, we simulated the imaging process according to the noise model and compared the simulated data to measured data. We estimated the values of the parameters μ_G_ and σ_G_ by using so-called dark images recorded at 0 mW excitation laser power. Because the Gaussian-distributed part of the noise is independent of the signal, μ_G_ and σ_G_ can be estimated as mean und standard deviation of dark images in which the ground-truth signal and thus the signal-dependent Poisson-distributed noise term equals zero. By using this parametrization, the simulated data show very good congruence (coefficient of determination *R*^2^ = 0.99) with the measured data (Figure 3).

### Improvements of the BM3D algorithm

An overview of algorithm steps improved and optimized for use in 2PM is given in Figure 2. In the following, we describe in detail two major changes and present the results of their evaluation.

#### Noise-adaptive threshold for block-matching

To identify blocks that are similar to the currently processed reference block, the threshold *Th*_BM_ is used by the BM3D algorithm to determine if two blocks are similar or not. For the first level, *Th*_BM_ is set to:

[4]
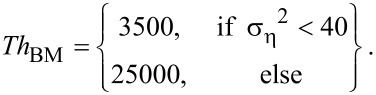


The following parameterization is used for the second level:

[5]
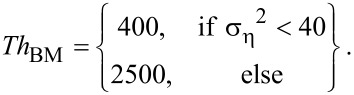


We modified this threshold in a way that allows for a more differentiated adaptation to the noise variance. The proposed parameterization is based on an estimation of the mean squared pixel difference between two noisy blocks with identical ground-truth signal. Assuming that the noise signals of such two blocks are pairwise uncorrelated, the Bienaymé formula [20] can be used to calculate the variance of the sum of both noise signals. Therefore, we can describe the variance of the pixel difference, σ_ε_^2^, caused by noise between these blocks:

[6]
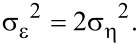


Because we also attempt to group blocks whose ground-truth signal is similar but not equal, we define an upper limit *Th*_offset_ for the variance of the pixel difference between the ground-truth signals of blocks, which are considered as similar. The sum of σ_ε_^2^ and *Th*_offset_ then gives us the variance of the pixel difference between noisy blocks with similar ground-truth signal. This value is used as threshold for the block-matching:

[7]
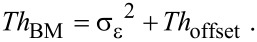


Based on tests with typical 2PM data sets (see Supporting Information File 1) we propose *Th*_offset_ = 900 at the first level. So *Th*_BM_ equals:

[8]
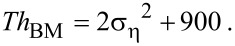


At the second level, a basic estimation of the ground-truth signal is used for block-matching. Because the noise has already been reduced, the noise-dependent term of the threshold has to be reduced as well. Tests showed that, for typical intravital 2PM images, the variance of the noise was reduced to approximately 30%. Concluding from these tests, we propose for the second level:

[9]
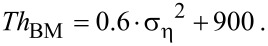


**Figure 4:**
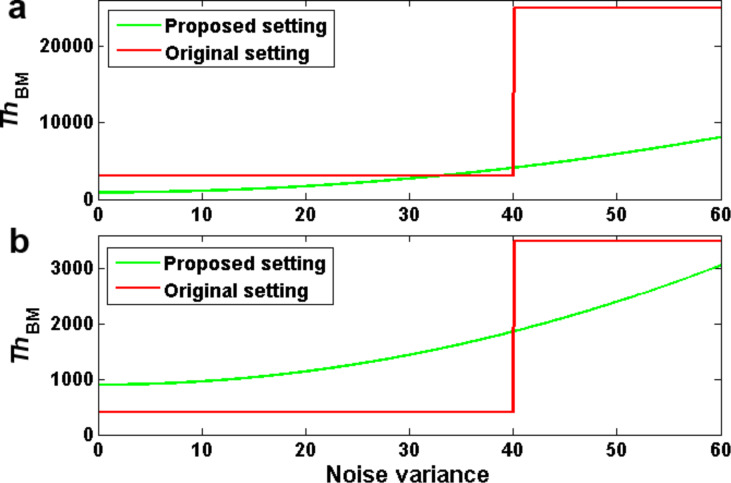
Parameterization of the block-matching: a) at the first level, b) at the second level.

As shown in Figure 4, our proposed modifications lead to substantial changes of the parameterization, especially for very noisy images.

#### Data-adaptive noise suppression

The suppression of noise is done by hard thresholding in a sparse representation domain (e.g., the wavelet domain). Instead of using a threshold that solely depends on σ_η_^2^, we propose a threshold that depends on the data as well. For this purpose, we adapted the threshold introduced by Chang et al. [21] to the BM3D algorithm: the threshold *Th*_Bayes_, which derives from a Bayesian network, is calculated separately for each subband of wavelet transformed blocks:

[10]
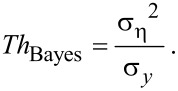


The standard derivation σ_y_ of the ground-truth signal is estimated as described in Chang et al. [21]. The decomposition method applied by the original BM3D algorithm results in very small subbands that consist of only a few wavelet coefficients. An initial implementation of the modified algorithm revealed that such subbands lead to an unreliable estimation of σ_y_. We therefore propose the use of the non-standard decomposition (see [22]) that leads to larger subbands and thus to reliable estimations of σ_y_.

#### Quantitative evaluation of the adapted algorithm

**Figure 5:**
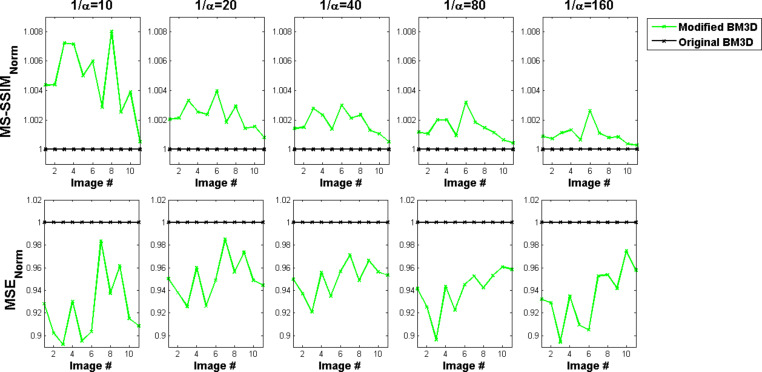
Quantitative evaluation of reconstructions by means of the original and the proposed version of the BM3D algorithm. Lower values of the parameter 1/α imply higher noise variances. The green curves show relative values as compared to the original BM3D algorithm (black curves). Higher MS-SSIM-values and lower MSE values represent better results. The measurements show that our modified version of the BM3D algorithm generally improves image quality independent of the amount of noise (depending on α) and the type of image (see Supporting Information File 1, Figure S2).

We applied both the original BM3D algorithm and our modified BM3D version to noisy test images with known ground truth. A quantitative evaluation was achieved by comparing ground truth and reconstructions of the original and our modified version in means of MSE (mean squared error) and MS-SSIM (multiscale structural similarity [23]) values (see Experimental). As shown in Figure 5, both metrics, MSE and MS-SSIM state improved reconstruction results of our proposed version compared to the original BM3D algorithm.

**Figure 6:**
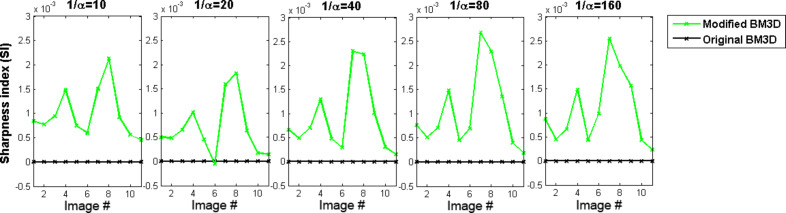
Sharpness index of reconstructions by the original and the modified version of the BM3D algorithm. Lower values of the parameter 1/α imply higher noise variances. Positive values for almost all images tested indicate an improved sharpness of the modified BM3D algorithm as compared to the original version. Note that this effect is independent of the amount of noise (depending on 1/α) contained in the image.

A further quantitative evaluation was done by calculating the sharpness index (SI) of the reconstructions (see Experimental). While this metric is only rarely applied to evaluations of image reconstructions, it is often used for automated focusing of light microscopes [24]. The SI values largely differ between the reconstructions of the original BM3D algorithm and our modified version (Figure 6). The increased SI values indicate that our modifications lead to a reduced flattening of edges in the reconstructed images.

#### Qualitative evaluation of the adapted algorithm

**Figure 7:**
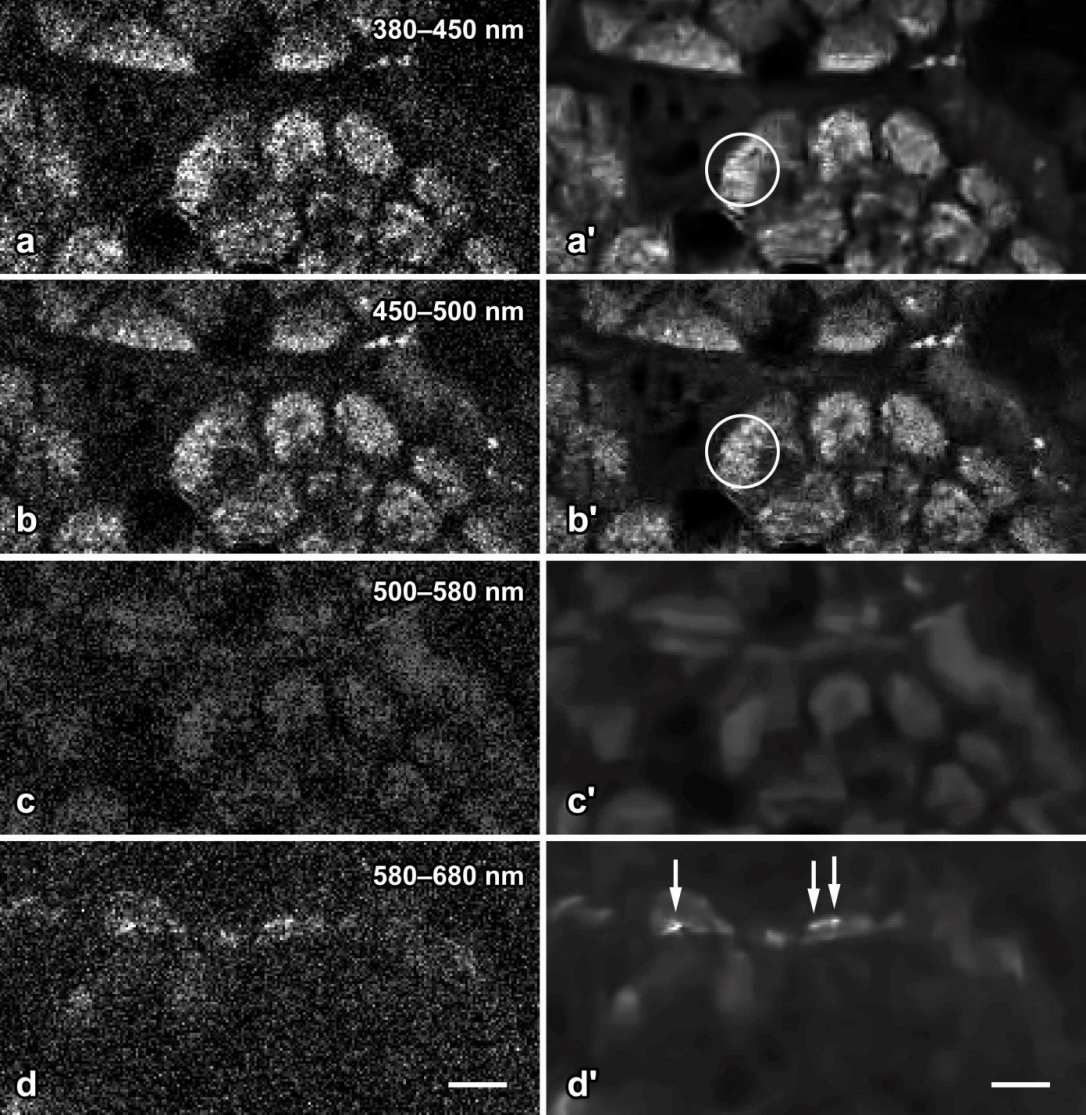
Epithelial cells and quantum dot nanoparticles of the murine gut mucosa in intravital 2-photon microscopy. The eight images correspond to the same field of view; raw data are shown in the left column (a–d), and the corresponding denoised images in the right (a′–d′). Nonlinear excitation of tissue and nanoparticle fluorescence was carried out 730 nm. The emitted light was split to four spectral channels, separated by dicroic mirrors at 450, 500 and 580 nm. The modified BM3D algorithm successfully reduces shot noise, but preserves fine structural details in the apical cytoplasm of the cells (encircled in a′ and b′). Quantum dot nanoparticles (arrows in d′) adhere to the apical surface of the cells and emit in channel 4 only. Denoising by the modified BM3D algorithm considerably facilitates the perception of the nanoparticles by the human observer (compare d to d′) and allows for automated image analysis to be applied to denoised 2PM images. Bar = 5 µm.

**Figure 8:**
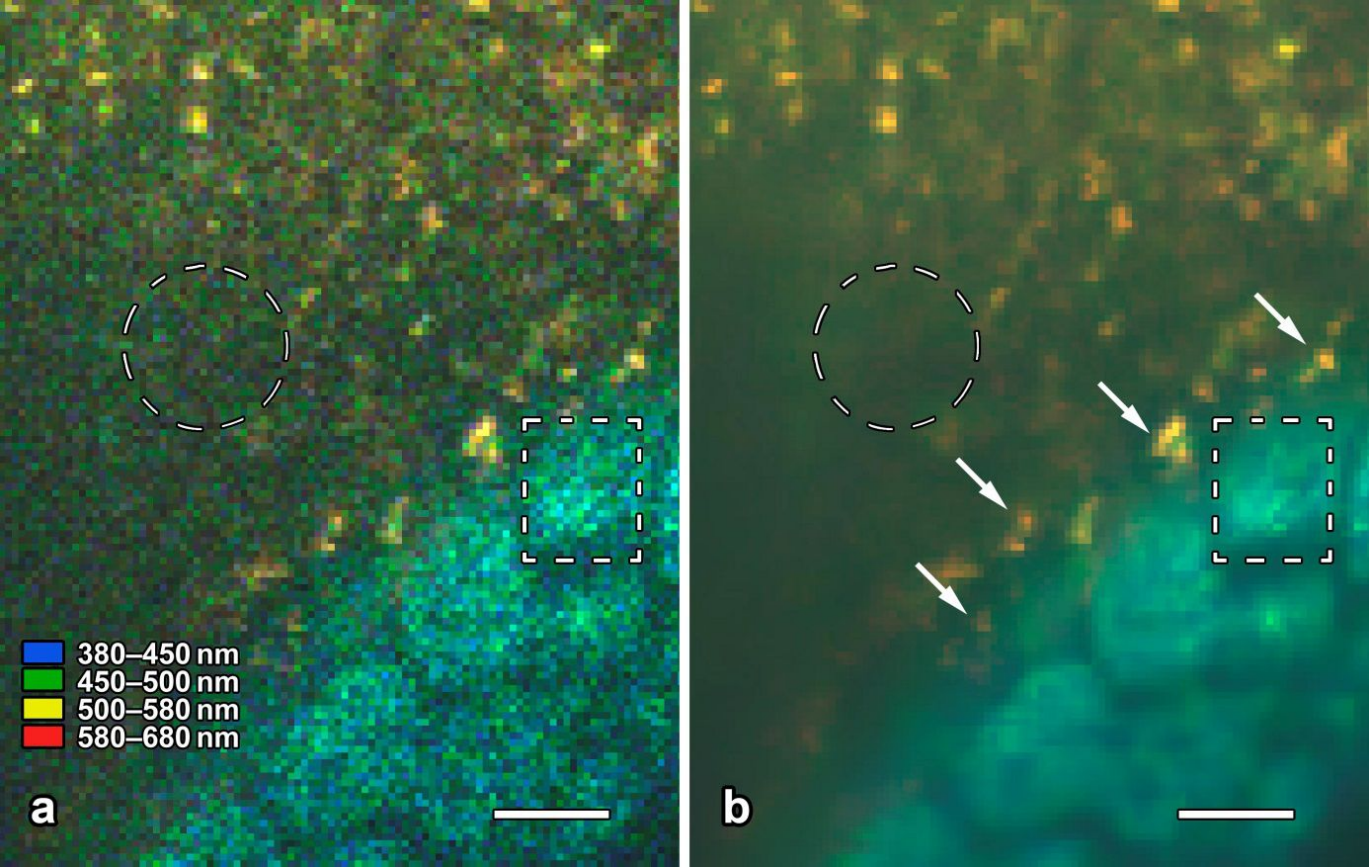
Intravital 2-photon microscopy of the gut mucosa (lower right corner) and quantum dot nanoparticles (yellow). Some of the nanoparticles adhere to mucus (upper third in a and b), some others adhere to the apical membrane of the epithelial cells (arrows in b). The grainy structure in the lumen (encircled in a) represents photon shot noise only; it is completely removed by the modified BM3D algorithm (b). In contrast, the structure of densely packed mitochondria is preserved in regions where the optical section runs through the apical cytoplasm of the cells (rectangle in b). Bar = 5 µm. The display colors selected for the 4 spectral channels are listed in a and almost meet the native color perception of the human eye.

In a series of qualitative evaluations, reconstructed images of the original and our modified version were compared by three human specialists for intravital 2PM (A.G., A.K., G.H.). All of them stated that the modified version we propose led to reduced artefacts and a better preserved representation of nanoparticles and other fine structures compared to the original BM3D algorithm (Figure 7 and Figure 8). Furthermore, in good agreement with the quantitative evaluation with regard to SI, they noticed an overall sharper appearance of reconstructions by the proposed version.

## Conclusion

BM3D, a state of the art algorithm for denoising, was evaluated regarding its suitability for images generated by intravital 2PM of living tissues that contain nanoparticles. The original BM3D algorithm produced better results than other advanced denoising algorithms, but still generated some unwanted artefacts and partly removed representations of fine structures. We developed different approaches to adapt the algorithm not only to the specific noise characteristics of 2PM data but also to the characteristics of the underlying ground-truth signal. Our findings show that these adaptions preserve representations of nanoparticles and other fine structures and reduce reconstruction artefacts. Based on a qualitative evaluation we conclude that our proposed version is more suitable for biomedical analysis of intravital 2PM images than the original BM3D algorithm.

The proposed denoising method not only facilitates the perception of structures in regard to a human rating and MS-SSIM values but also increases the SNR to a multiple. This approach will allow us to reduce excitation power to a fractional amount while, by means of our proposed denoising method, the required image quality is maintained. Because a reduced excitation power implies reduced phototoxity, this allows 2PM images to be taken at higher frequencies and/or over longer periods of time. Our method is applicable to other imaging modalities as well, provided the specific noise characteristics are known and taken into account.

## Experimental

### Setup for intravital imaging in mice

**Figure 9:**
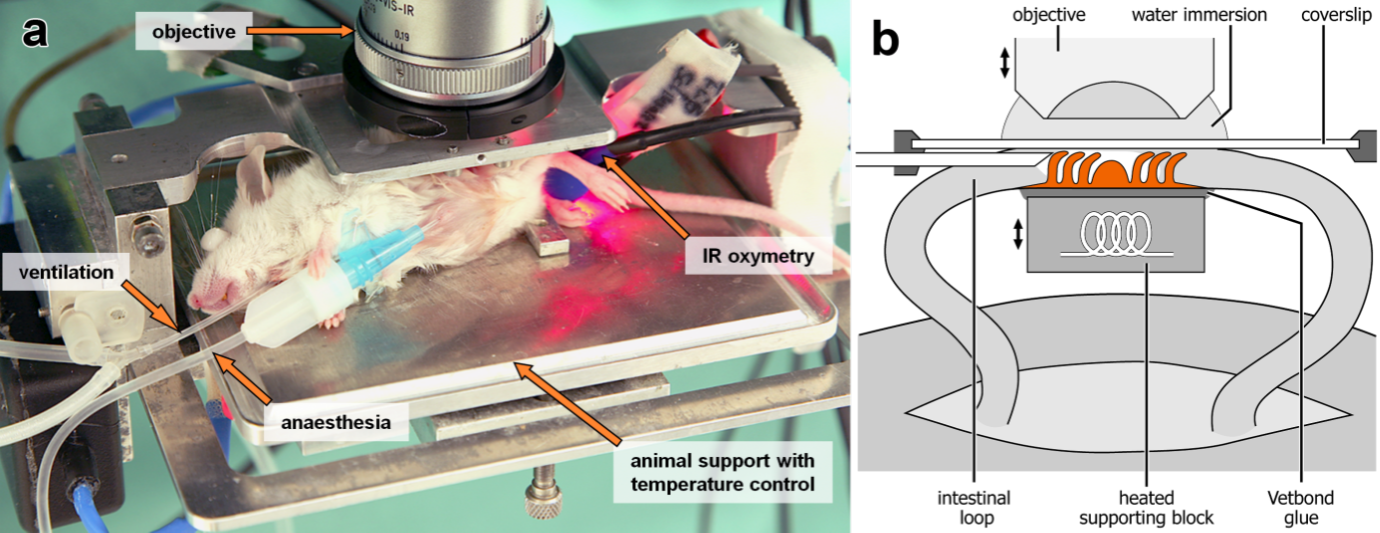
a) Anaesthetized Balb/c mouse on a homeothermic table with an exteriorized ileal loop. b) Schematic diagram of the chamber for intravital 2PM imaging.

Balb/c mice (Charles River, Sulzfeld, Germany), about 10 weeks old, were kept under standard animal house conditions and had free access to water and food. The mice were anaesthetized by a mixture of Fentanyl (Bayer, Leverkusen, Germany), Medetomidin (Pfizer, Karlsruhe, Germany) and Midazolam (Curamed, Karlsruhe, Germany), injected intraperitoneally. As described previously [1], the abdominal cavity was opened surgically and an isolated loop of the small intestine was glued onto a warmed supporting block. The gut wall was sliced and the mucosa was carefully pressed to a fixed microscopic cover slip to dampen movement artefacts (Figure 9). The gut segment was constantly moisturized with pre-warmed saline, and the body core temperature was maintained at 37 °C by using a homeothermic table. The mucosa was steadily perfused, as seen by an erythrocyte movement phenomenon, and the tissue remained fully motile with no evidence of decreased viability for experiments lasting up to 8 h. The animal experiments were approved by the local government (Ministerium für Umwelt, Naturschutz und Landwirtschaft Schleswig-Holstein, Germany, V742-72241.122).

### Preparation and properties of nanoparticles

CdSe/CdS/ZnS-core/shell/shell quantum dots (QDs) used in this study were provided by the Center for Applied Nanotechnology, CAN GmbH, Germany (CANdots, Series A). These nanocrystals are originally dispersed in a nonpolar organic solvent. To allow for bioapplications they were transferred into the aqueous phase by encapsulation within amphiphilic shells of crosslinked poly(isoprene)-*block*-poly(ethylene glycol) (PI-*b*-PEG), following a procedure described in detail previously [25–27]. The outer PEG-blocks were exo-functionalized with carboxy groups. After phase transfer, the PI-*b*-PEG-encapsulated QDs had a hydrodynamic diameter of ca. 25 nm, as determined by dynamic light scattering (DLS). The spectral position of the excitonic emission band was located at ca. 585 nm (FWHM: ca. 32 nm), and the photoluminescence quantum yield was around 20–30%.

### Setup of the 2-photon microscope

Intravital 2PM was done by using a JenLab DermaInspect 101 system (JenLab, Jena, Germany) equipped with a tunable femtosecond Ti:sapphire laser (Spectra Physics, Mountain View, CA, USA) and 40×/1.2 and 20×/1.0 water immersion objective lenses (Zeiss, Jena, Germany). The excitation wavelength was tuned between 710 and 920 nm and typically set to 730 nm, a wavelength which is known to mainly excite NAD(P)H [28]. Emission was detected in up to four separate spectral channels between 380 and 560 nm (for details see [29]). Digital images typically covered a field of 150 × 150 µm^2^ at a pixel size of 0.29 × 0.29 µm^2^.

#### Implementation

The algorithms were mainly implemented by using the Matlab programming language. To improve performance, the C++ language was used for some parts of the program code.

### Setup for noise measurements

Our noise measurements were done at identical settings of the 2PM intravital imaging (see above), thereby ensuring that the noise of intravital measurements and noise measurements had identical distribution and statistical properties. Autofluorescent plastic slides (type 92001, Chroma Technology Corp., Bellows Falls, VT, USA) which provide a homogeneous distribution of fluorophores were used as probes. We performed 24 measurements by step-wise increasing the laser beam power from 0.0 mW to 2.77 mW (measured below the objective lens).

### Setup for evaluation of denoising methods

Evaluation was split into a quantitative and a qualitative part. For each part, a separate set of test images was used.

For quantitative evaluation, high-quality images composed of 512 × 512 pixels were acquired by averaging 32 intensity measurements per pixel. Eleven high-quality images (see Supporting Information File 1, Figure S2) were selected from several hundred images we acquired. These 11 images were declared as ground-truth signal by definition. From these test images, we generated different noisy versions by means of the noise model, which was successfully validated before, using different parameterizations. The quantitative evaluation was finally done by comparing the reconstructions of the denoising methods with the ground-truth signal by means of MSE and MS-SSIM.

For qualitative evaluation, additional images from current biomedical investigations were selected (Figure 7 and Figure 8 show representative examples). As the ground-truth signal of the second test set is unknown, the evaluation was done with regard to the integrity and recognizability of relevant biological structures and the generation of unwanted reconstruction artefacts.

## Supporting Information

File 1 Additional experimental data.
