# Supplementary material for: Data-adaptive image-denoising for detecting and quantifying nanoparticle entry in mucosal tissues through intravital 2-photon microscopy
Source: Beilstein J Nanotechnol. 2014 Nov 6;5:2016–25. doi: 10.3762/bjnano.5.210 (PMC4273251; doi:10.3762/bjnano.5.210)
Supplement: File 1 — Additional experimental data. [file Beilstein_J_Nanotechnol-05-2016-s001.pdf]

Supporting Information

for

Data-adaptive image-denoising for detecting and quantifying nanoparticle entry in mucosal tissues through intravital 2-photon microscopy

Torsten Bölke[1], Lisa Krapf[2], Regina Orzekowsky-Schroeder[3], Tobias Vossmeyer[4], Jelena Dimitrijevic[4], Horst Weller[4], Anna Schüth[5], Antje Klinger[5], Gereon Hüttmann[2] and Andreas Gebert*[1]

Address: [1]University Hospital Jena, Friedrich Schiller University Jena, Institute of Anatomy II, Teichgraben 7, 07740 Jena, Germany; [2]University of Lübeck, Institute of Biomedical Optics, Peter-Monnik-Weg 4, 23562 Lübeck, Germany; [3]Olympus Winter & Ibe GmbH, R&D Optical Design, Kuehnstrasse 61, 22045 Hamburg, Germany; [4]University of Hamburg, Institute of Physical Chemistry, Grindelallee 117, 20146 Hamburg, Germany and [5]University of Lübeck, Institute of Anatomy, Ratzeburger Allee 160, 23538 Lübeck, Germany

Email: Andreas Gebert* - andreas.gebert@med.uni-jena.de

* Corresponding author

Additional experimental data

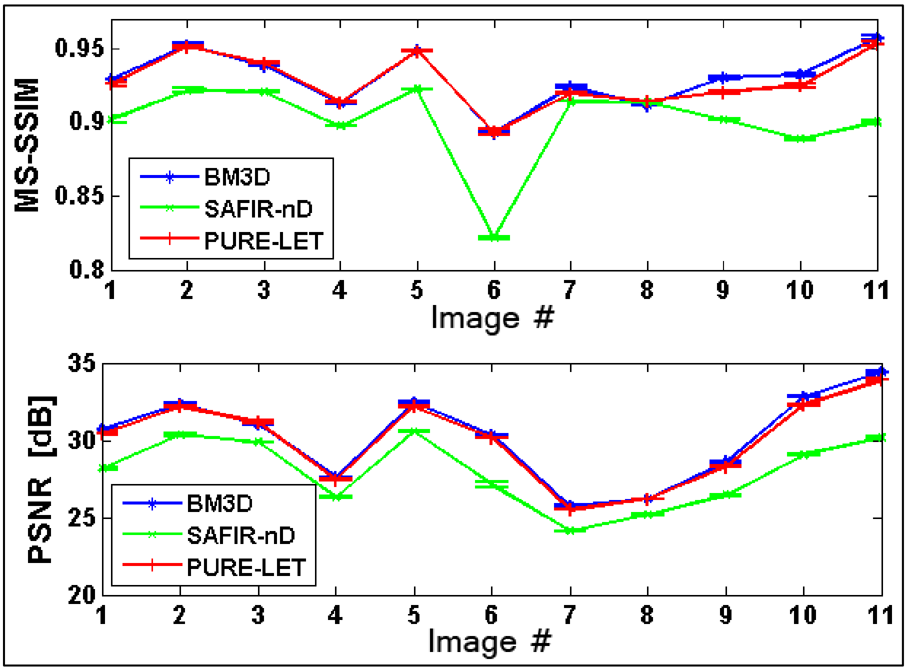

Figure S1: Quantitative evaluation of the denoising power of the BM3D, SAFIR-nD, and PURE-LET algorithms.

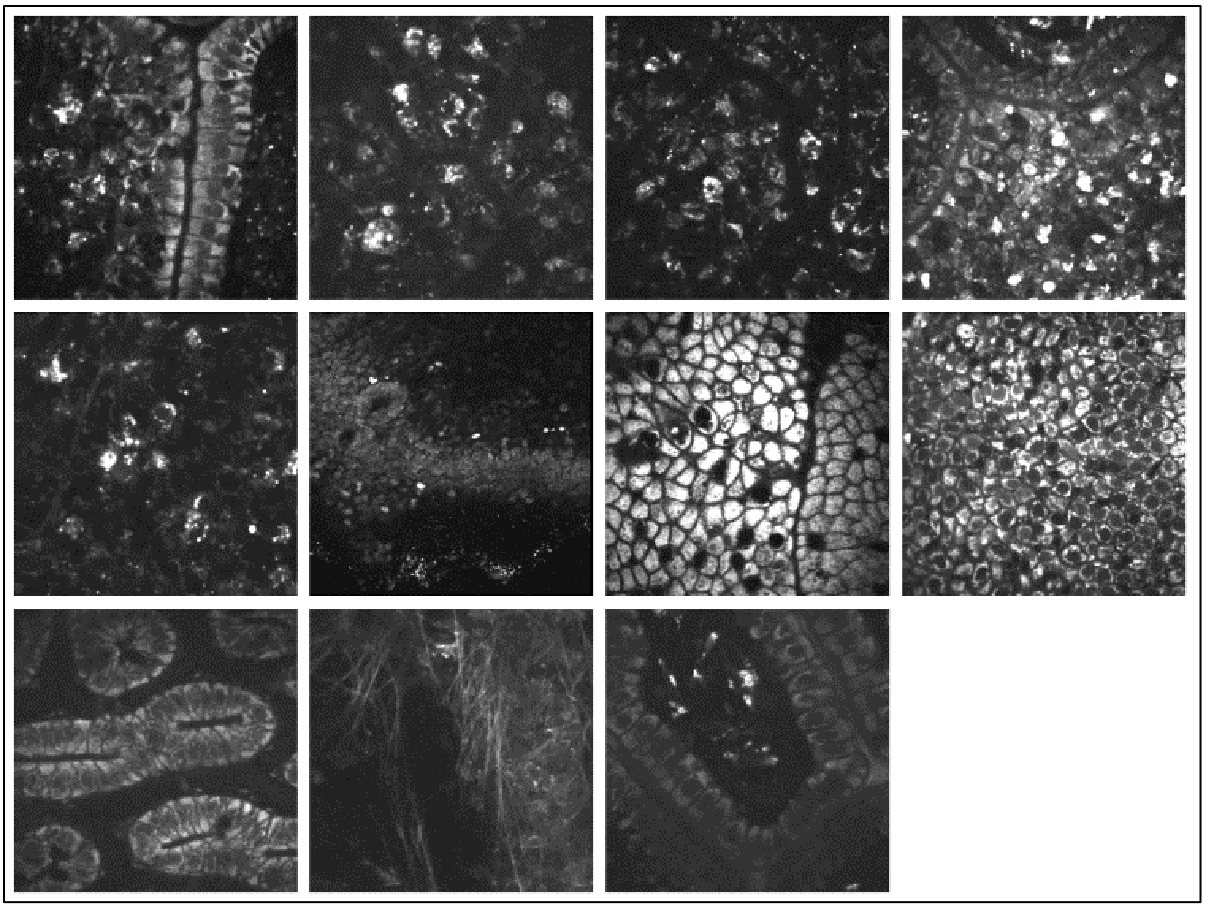

Figure S2: Set of 2PM images used as ground truth signal for quantitative evaluation of the different denoising methods.
